# Genome Wide Analysis of Differentially Expressed Genes in HK-2 Cells, a Line of Human Kidney Epithelial Cells in Response to Oxalate

**DOI:** 10.1371/journal.pone.0043886

**Published:** 2012-09-19

**Authors:** Sweaty Koul, Lakshmipathi Khandrika, Randall B. Meacham, Hari K. Koul

**Affiliations:** 1 Signal Transduction and Molecular Urology Laboratory-Program in Urosciences, Division of Urology/Department of Surgery, University of Colorado School of Medicine, Aurora, Colorado, United States of America; 2 University of Colorado Comprehensive Cancer Center, University of Colorado School of Medicine, Aurora, Colorado, United States of America; 3 Denver Veterans Affairs Medical Center, Denver, Colorado, United States of America; Children's Hospital Boston & Harvard Medical School, United States of America

## Abstract

Nephrolithiasis is a multi-factorial disease which, in the majority of cases, involves the renal deposition of calcium oxalate. Oxalate is a metabolic end product excreted primarily by the kidney. Previous studies have shown that elevated levels of oxalate are detrimental to the renal epithelial cells; however, oxalate renal epithelial cell interactions are not completely understood. In this study, we utilized an unbiased approach of gene expression profiling using Affymetrix HG_U133_plus2 gene chips to understand the global gene expression changes in human renal epithelial cells [HK-2] after exposure to oxalate. We analyzed the expression of 47,000 transcripts and variants, including 38,500 well characterized human genes, in the HK2 cells after 4 hours and 24 hours of oxalate exposure. Gene expression was compared among replicates as per the Affymetrix statistical program. Gene expression among various groups was compared using various analytical tools, and differentially expressed genes were classified according to the Gene Ontology Functional Category. The results from this study show that oxalate exposure induces significant expression changes in many genes. We show for the first time that oxalate exposure induces as well as shuts off genes differentially. We found 750 up-regulated and 2276 down-regulated genes which have not been reported before. Our results also show that renal cells exposed to oxalate results in the regulation of genes that are associated with specific molecular function, biological processes, and other cellular components. In addition we have identified a set of 20 genes that is differentially regulated by oxalate irrespective of duration of exposure and may be useful in monitoring oxalate nephrotoxicity. Taken together our studies profile global gene expression changes and provide a unique insight into oxalate renal cell interactions and oxalate nephrotoxicity.

## Introduction

Oxalate is a metabolic end product that is freely filtered at the glomerulus, undergoes bi-directional transport in the renal tubules, and is excreted primarily by the kidney [1]–[3]. The most common pathological condition involving oxalate is the formation of calcium oxalate stones in the kidney [4]. While very high levels of urinary oxalate are observed only in subjects with primary hyperoxaluria, a majority of idiopathic kidney stone patients only show a mild elevation in urinary oxalate [5]–[9] In addition several other conditions associated with oxalate deposits are: renal cysts in acquired renal cystic disease [7], proliferating cells in the kidney [8], hyperplasic thyroid glands [9], and benign neoplasm of the breast [10], [11]. These considerations suggest that the pathological deposition of calcium oxalate is more complex than a simple physical precipitation of calcium oxalate crystals. In 1994, we were the first group to note that oxalate renal cell interactions involved alterations in gene expression [12]. Over the past two decades, studies [13]–[23] have demonstrated that oxalate interactions with renal epithelial cells result in a program of events, including changes in gene expression and cell dysfunction, consistent with cellular stress. Studies from our laboratory demonstrated that oxalate induced changes in renal cells (viz. re-initiation of DNA synthesis) are inhibited by inhibitors of transcription and translation, indicating that the cellular response to oxalate toxicity is dependent on new gene expression and protein synthesis [24]. Moreover, cells of the renal tubular epithelium are exposed to an environment with variable and elevated concentrations of the oxalate and must be able to adapt to oxalate stress. Indeed we have shown that many signal transduction pathways, including p38 MAPK and JNK, are activated in renal epithelial cells in response to oxalate and COM crystals [14], [24]. However, the genetic response of renal epithelial cells to oxalate exposure remains ambiguous.

HK-2 cells are a line of human proximal tubular epithelial cells immortalized by using the E6/E7 genes of human papilloma virus [HPV 16] [25]. These cells retain the characteristics of proximal renal tubular epithelium and have been used successfully as an in vitro model system to represent the human kidney epithelial cells. Previous studies have identified a number of stress and inflammation related genes whose expression is increased in renal epithelial cell cultures responding to oxalate [12], [18], [22]. However, these studies provide limited information on the gene expression program orchestrated in renal epithelial cells in response to increased levels of oxalate. In general it is believed that the effect of oxalate in renal epithelial cells is mediated, at least in part, through the regulation of gene transcription. Therefore to gain insights into molecular events associated with oxalate nephrotoxicity we used HK2 cells and cDNA microarray technology, which allows for the simultaneous analysis of multiple gene expression patterns, to evaluate changes in the global gene expression in renal epithelial cells in response to oxalate [26]. We used Affymetrix hg_u133_plus 2 gene arrays comprised of 54000 probe sets and 1,300,000 distinct oligonucleotide features representing 47,000 transcripts and variants including 38,500 well-characterized human genes. Preliminary studies [27], presented in this report, shows that exposure to oxalate elicits a specific gene expression response: oxalate exposure regulates expression of a much larger number of genes than previously thought. We also demonstrated that in addition to upregulating gene expression oxalate exposure results in the down-regulation of expression of a large set of genes. Moreover, our analysis of this gene expression data also reveals an array of twenty new genes that are differentially regulated by oxalate and could be useful for monitoring oxalate nephrotoxicity. Our studies provide a large amount of the data that may help improve our understanding of the pathogenesis associated with hyperoxaluria in general, and more specifically, with oxalate renal cell interactions.

## Results

### Treatment with oxalate causes unprecedented changes in gene expression in renal epithelial cells

**Figure 1:**
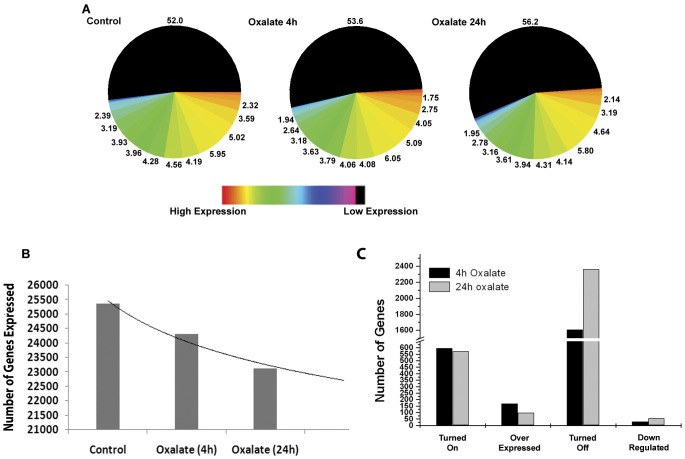
Exposure of HK-2 cells to oxalate results in changes in global gene expression. (A) Levels of gene expression are color coded to depict high to low expression and the numbers represent percentages of genes at each expression level. Representative images from duplicate experiments are shown. (B) Quantitative representation of translational suppression following exposure to Oxalate. (C) Quantitative analysis of differential gene expression in HK2 cells following oxalate exposure. Each data point represents mean of two individual experiments.

Affymetrix gene chip analysis for changes in gene expression in HK-2 cells upon oxalate exposure identified global changes in the number of genes that are differentially expressed (Figure 1A). We analyzed changes in gene expression upon short term, 4 hours, and long term, 24 hours, exposure of HK-2 cells to oxalate. Of the 47,000 gene expression transcripts and variants analyzed on the gene chip, 26211 transcripts are expressed in the control compared to 25107 transcripts upon short term (4 h) and 23935 transcripts upon long term exposure (24 h) to oxalate (Fig. 1B). These results show that oxalate exposure to the renal cells is associated with the transcriptional silencing of over 8.55 of all transcripts. These results reveal that oxalate exposure, in addition to turning on gene expression, is associated with the significant suppression of gene expression in renal epithelial cells.

### Effects of oxalate on the modulation of gene expression in HK2 cells are selective

Even though there is a major reduction in the number of genes that were expressed upon oxalate exposure, the results presented in figure 1C indicate that about 600 genes that were expressed upon oxalate exposure were not expressed in the control cells at all. In addition, these results show over 150 genes are over-expressed in response to oxalate exposure. New genes are turned on in as little as 4 hours exposures to oxalate and are observed even after 24 hours of oxalate exposure. The results presented in figure 1C and table 1 show that oxalate exposure results in the complete silencing of some 1104 transcripts in as little as 4 hours and 2,276 transcripts in 24 hours of exposure. Taken together these results demonstrate the complex nature of oxalate interactions with renal cells and demonstrate that oxalate selectively turns on some sets of genes, while selectively turning off others. This data also suggests that oxalate induced changes seen in renal epithelial cells may be driven by the modulation of gene expression.

**Table 1:** Changes in gene expression upon exposure of HK-2 cells to oxalate.

Treatment	Genes Absent	Genes Strongly Expressed	Genes Mildly Expressed
Control	28464	25367	844
Oxalate (4 h)	29568	24312	795
Oxalate (24 h)	30780	23102	883

### Oxalate differentially regulates the expression of specific genes associated with molecular function, biological processes as well as cellular components

**Figure 2:**
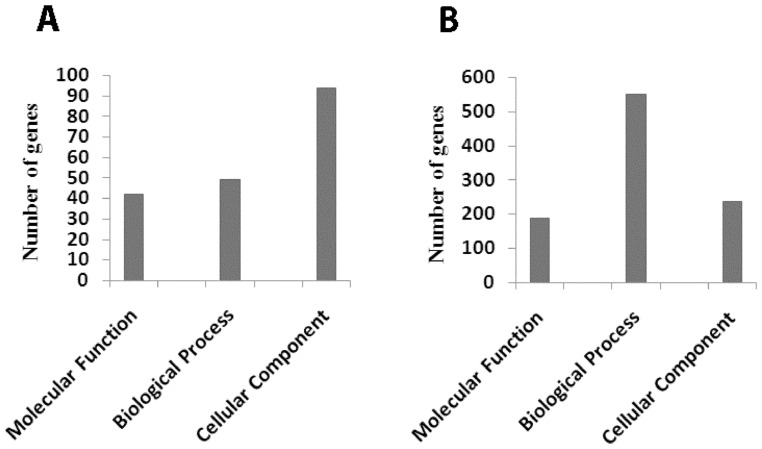
Exposure to oxalate modulates signaling pathways in HK-2 cells. Changes in the expression of genes involved in various signaling pathways upon exposure of HK-2 cells to oxalate, as determined by Affymetrix gene chip analysis. (A) Changes in gene expression of genes involved in signal transduction pathways after exposure to oxalate. (B) Heat map and cluster diagram represent changes in expression of genes that constitute the Mitogen Activated Protein Kinase (MAPK) pathway. Each data point is representative of the median expression level of two independent gene chip arrays. The Cluster diagram and dendogram was generated using BRB-Array Tools software with a minimum fold change less than 20% of expression data and have at least a 1.5 fold change in either direction from the gene's median expression value.

Genes that were either up or down regulated in the oxalate treated cells were categorized based on gene ontology using GenMAPP software. The results presented in Fig. 2A and Table 2 lists genes which are up-regulated greater than fivefold in HK2 cells in response to oxalate exposure. When categorized based on gene ontology (using GenMAPP software), we observed that of the 6,725 genes associated with molecular functions, oxalate exposure resulted in the upregulation of 152 genes. Similarly, of the 16,467 genes associated with biological processes oxalate exposure resulted in the upregulation of 105 genes; and among the 10,435 genes associated with various cellular components oxalate exposure results in the upregulation of 94 of them.

**Table 2:** Genes that show an average change more than 5 fold up-regulation compared to control (p value<0.05) upon oxalate exposure grouped based on gene ontology using GenMAPP.

2.1 Molecular Function								
Go ID	GO Name	Genes	Genes	Genes	% Changed	% Present	Z Score	p Value
		Changed	Measured	in GO				
30528	transcription regulator activity	15	1012	1398	1.482213	72.38913	3.664	0
16941	natriuretic peptide receptor activity	1	1	1	100	100	12.651	0.001
19955	cytokine binding	3	44	87	6.818182	50.57471	5.244	0.003
4888	transmembrane receptor activity	8	429	1294	1.864802	33.15302	3.347	0.003
4907	interleukin receptor activity	2	17	32	11.76471	53.125	5.854	0.004
19965	interleukin binding	2	20	38	10	52.63158	5.344	0.004
42610	CD8 receptor binding	1	1	1	100	100	12.651	0.006
3700	transcription factor activity	10	630	936	1.587302	67.30769	3.182	0.007
4872	receptor activity	11	752	1799	1.462766	41.801	3.047	0.007
42609	CD4 receptor binding	1	2	2	50	100	8.89	0.008
4184	lysine carboxypeptidase activity	1	1	2	100	50	12.651	0.009
5142	interleukin-11 receptor binding	1	1	1	100	100	12.651	0.009
51117	ATPase binding	1	2	3	50	66.66666	8.89	0.009
4126	cytidine deaminase activity	1	2	5	50	40	8.89	0.009
4896	hematopoietin/interferon-class (D200-domain)	2	33	54	6.060606	61.11111	3.984	0.01
	cytokine receptor activity							
45029	UDP-activated nucleotide receptor activity	1	2	2	50	100	8.89	0.011
16494	C-X-C chemokine receptor activity	1	3	10	33.33333	30	7.214	0.011
5006	epidermal growth factor receptor activity	1	3	7	33.33333	42.85714	7.214	0.011
19958	C-X-C chemokine binding	1	3	11	33.33333	27.27273	7.214	0.011
19957	C-C chemokine binding	1	4	16	25	25	6.208	0.012
16493	C-C chemokine receptor activity	1	4	16	25	25	6.208	0.012
60089	molecular transducer activity	13	1071	2228	1.213819	48.07002	2.603	0.012
4871	signal transducer activity	13	1071	2228	1.213819	48.07002	2.603	0.012
4911	interleukin-2 receptor activity	1	2	3	50	66.66666	8.89	0.015
19976	interleukin-2 binding	1	2	3	50	66.66666	8.89	0.015
43533	inositol 1\,3\,4\,5 tetrakisphosphate binding	1	2	2	50	100	8.89	0.016
43178	alcohol binding	1	2	2	50	100	8.89	0.016
3704	specific RNA polymerase II transcription	2	31	41	6.451613	75.60976	4.139	0.016
	factor activity							
46592	polyamine oxidase activity	1	2	2	50	100	8.89	0.017
46983	protein dimerization activity	5	226	294	2.212389	76.87075	3.078	0.017
8528	peptide receptor activity\, G-protein coupled	2	32	115	6.25	27.82609	4.06	0.018
1653	peptide receptor activity	2	32	115	6.25	27.82609	4.06	0.018
43548	phosphoinositide 3-kinase binding	1	4	4	25	100	6.208	0.019
16647	oxidoreductase activity\, acting on the CH-NH	1	3	3	33.33333	100	7.214	0.02
	group of donors\, oxygen as acceptor							
15065	uridine nucleotide receptor activity	1	3	3	33.33333	100	7.214	0.02
4383	guanylate cyclase activity	1	5	9	20	55.55556	5.518	0.02
3707	steroid hormone receptor activity	2	34	59	5.882353	57.62712	3.912	0.021
8022	protein C-terminus binding	2	40	45	5	88.88889	3.532	0.022
19911	structural constituent of myelin sheath	1	3	3	33.33333	100	7.214	0.023
4370	glycerol kinase activity	1	4	4	25	100	6.208	0.023
4879	ligand-dependent nuclear receptor activity	2	36	62	5.555555	58.06452	3.775	0.024
5244	voltage-gated ion channel activity	3	99	181	3.030303	54.69613	3.066	0.024
5179	hormone activity	2	39	112	5.128205	34.82143	3.59	0.025
8060	ARF GTPase activator activity	1	4	4	25	100	6.208	0.027
1637	G-protein chemoattractant receptor activity	1	7	27	14.28571	25.92593	4.604	0.03
4950	chemokine receptor activity	1	7	27	14.28571	25.92593	4.604	0.03
1948	glycoprotein binding	1	4	6	25	66.66666	6.208	0.032
5521	lamin binding	1	5	5	20	100	5.518	0.032
16564	transcriptional repressor activity	4	194	236	2.061856	82.20339	2.578	0.032
32395	MHC class II receptor activity	1	5	22	20	22.72727	5.518	0.033
19956	chemokine binding	1	8	29	12.5	27.58621	4.279	0.036
19899	enzyme binding	4	209	250	1.913876	83.6	2.403	0.038
5261	cation channel activity	3	129	246	2.325581	52.43903	2.48	0.044
42826	histone deacetylase binding	1	10	11	10	90.90909	3.777	0.048
5328	neurotransmitter\:sodium symporter activity	1	8	20	12.5	40	4.279	0.049
5326	neurotransmitter transporter activity	1	8	22	12.5	36.36364	4.279	0.049
5102	receptor binding	6	418	742	1.435407	56.33423	2.162	0.049

2.2 Biological Process								
30854	positive regulation of granulocyte differentiation	1	1	1	100	100	12.651	0.004
30852	regulation of granulocyte differentiation	1	1	2	100	50	12.651	0.004
16554	cytidine to uridine editing	1	1	2	100	50	12.651	0.005
18992	germ-line sex determination	1	1	1	100	100	12.651	0.005
7542	primary sex determination\, germ-line	1	1	1	100	100	12.651	0.005
19100	male germ-line sex determination	1	1	1	100	100	12.651	0.005
7538	primary sex determination	1	1	2	100	50	12.651	0.005
50862	positive regulation of T cell receptor signaling pathway	1	1	2	100	50	12.651	0.006
7422	peripheral nervous system development	2	16	26	12.5	61.53846	6.053	0.007
30219	megakaryocyte differentiation	1	1	3	100	33.33333	12.651	0.009
281	cytokinesis after mitosis	1	3	2	33.33333	150	7.214	0.01
50857	positive regulation of antigen receptor-mediated	1	2	4	50	50	8.89	0.013
	signaling pathway							
16553	base conversion or substitution editing	1	3	3	33.33333	100	7.214	0.014
51235	maintenance of localization	2	27	34	7.407407	79.41177	4.495	0.014
30321	transepithelial chloride transport	1	2	2	50	100	8.89	0.015
2763	positive regulation of myeloid leukocyte	1	5	5	20	100	5.518	0.015
	differentiation							
2521	leukocyte differentiation	3	81	128	3.703704	63.28125	3.545	0.016
30851	granulocyte differentiation	1	3	7	33.33333	42.85714	7.214	0.017
45885	positive regulation of survival gene product activity	1	3	6	33.33333	50	7.214	0.018
30238	male sex determination	1	4	7	25	57.14286	6.208	0.018
6182	cGMP biosynthetic process	1	5	12	20	41.66667	5.518	0.02
51282	regulation of sequestering of calcium ion	1	3	4	33.33333	75	7.214	0.021
51283	negative regulation of sequestering of calcium ion	1	3	4	33.33333	75	7.214	0.021
51209	release of sequestered calcium ion into cytosol	1	3	4	33.33333	75	7.214	0.021
6381	mRNA editing	1	4	6	25	66.66666	6.208	0.022
16556	mRNA modification	1	4	6	25	66.66666	6.208	0.022
22414	reproductive process	4	165	277	2.424242	59.56679	2.972	0.022
45773	positive regulation of axon extension	1	4	5	25	80	6.208	0.023
45604	regulation of epidermal cell differentiation	1	4	7	25	57.14286	6.208	0.024
6357	regulation of transcription from RNA	6	371	474	1.617251	78.27004	2.486	0.024
	polymerase II promoter							
45892	negative regulation of transcription\, DNA-dependent	4	181	218	2.209945	83.02752	2.745	0.027
7567	parturition	1	7	9	14.28571	77.77778	4.604	0.03
2376	immune system process	7	489	937	1.431493	52.18783	2.335	0.03
16547	RNA editing	1	6	7	16.66667	85.71429	5.005	0.031
19233	sensory perception of pain	1	7	15	14.28571	46.66667	4.604	0.032
6665	sphingolipid metabolic process	2	47	60	4.255319	78.33334	3.179	0.033
46716	muscle maintenance	1	6	6	16.66667	100	5.005	0.036
51208	sequestering of calcium ion	1	5	6	20	83.33334	5.518	0.037
46068	cGMP metabolic process	1	8	16	12.5	50	4.279	0.037
50856	regulation of T cell receptor signaling pathway	1	6	9	16.66667	66.66666	5.005	0.038
46928	regulation of neurotransmitter secretion	1	7	8	14.28571	87.5	4.604	0.038
45595	regulation of cell differentiation	3	113	149	2.654867	75.83893	2.767	0.038
42994	cytoplasmic sequestering of transcription factor	1	7	10	14.28571	70	4.604	0.041
6072	glycerol-3-phosphate metabolic process	1	7	8	14.28571	87.5	4.604	0.041
51094	positive regulation of developmental process	2	57	62	3.508772	91.93549	2.783	0.041
6688	glycosphingolipid biosynthetic process	1	7	11	14.28571	63.63636	4.604	0.042
45682	regulation of epidermis development	1	7	9	14.28571	77.77778	4.604	0.042
6882	zinc ion homeostasis	1	6	6	16.66667	100	5.005	0.044
50854	regulation of antigen receptor-mediated	1	7	11	14.28571	63.63636	4.604	0.044
	signaling pathway							
51238	sequestering of metal ion	1	7	9	14.28571	77.77778	4.604	0.045
45039	protein import into mitochondrial inner membrane	1	6	6	16.66667	100	5.005	0.046
42308	negative regulation of protein import into nucleus	1	8	12	12.5	66.66666	4.279	0.048
42992	negative regulation of transcription factor import	1	8	12	12.5	66.66666	4.279	0.048
	into nucleus							
1837	epithelial to mesenchymal transition	1	10	5	10	200	3.777	0.048
16575	histone deacetylation	1	10	13	10	76.92308	3.777	0.048
32501	multicellular organismal process	19	2030	3635	0.93596	55.84594	2.006	0.048
7530	sex determination	1	9	18	11.11111	50	4.008	0.049
3	reproduction	5	318	515	1.572327	61.74757	2.192	0.049

2.3 Cellular component								
5886	plasma membrane	15	1205	2017	1.244813	59.74219	2.925	0.007
5731	nucleolus organizer region	1	1	1	100	100	12.651	0.009
30874	nucleolar chromatin	1	1	1	100	100	12.651	0.009
5887	integral to plasma membrane	10	684	1209	1.461988	56.57568	2.893	0.01
31226	intrinsic to plasma membrane	10	690	1221	1.449275	56.51105	2.863	0.01
9986	cell surface	3	94	160	3.191489	58.75	3.187	0.018
792	heterochromatin	2	30	35	6.666667	85.71429	4.221	0.019
145	exocyst	1	4	5	25	80	6.208	0.024
44459	plasma membrane part	12	966	1647	1.242236	58.6521	2.576	0.024
16328	lateral plasma membrane	1	7	7	14.28571	100	4.604	0.042
42719	mitochondrial intermembrane space protein	1	6	6	16.66667	100	5.005	0.046
	transporter complex							
242	pericentriolar material	1	8	6	12.5	133.3333	4.279	0.048
5622	intracellular	34	6724	8743	0.505651	76.90724	−1.959	0.048
1518	voltage-gated sodium channel complex	1	7	12	14.28571	58.33333	4.604	0.049
42613	MHC class II protein complex	1	8	37	12.5	21.62162	4.279	0.049

The results presented in Fig. 2B and Table 3 reveal a list of genes which were down-regulated greater than fivefold in HK2 cells in response to oxalate exposure. When categorized based on gene ontology (using GenMAPP software), we observed that of the 5,230 genes associated with molecular functions oxalate exposure resulted in the down-regulation of 189 genes. Similarly, of the 16,467 genes associated with biological processes oxalate exposure resulted in the down-regulation of 552 genes; and among the 11,905 genes associated with various cellular components oxalate exposure resulted in the down-regulation of 239 genes.

**Table 3:** Genes that show an average change more than 5 fold down-regulation compared to control (p value<0.05) upon oxalate exposure grouped based on gene ontology using GenMAPP.

3.1 Molecular Function								
Go ID	GO Name	Genes	Genes	Genes	% Changed	% Present	Z Score	p Value
		Changed	Measured	In GO				
4095	carnitine O-palmitoyl-	2	3	4	66.66666	75	7.244	0.002
	transferase activity							
16416	O-palmitoyltransferase activity	2	3	4	66.66666	75	7.244	0.002
16406	carnitine O-acyltransferase activity	2	5	6	40	83.33334	5.471	0.003
42805	actinin binding	2	3	4	66.66666	75	7.244	0.004
5522	profilin binding	2	5	5	40	100	5.471	0.007
4527	exonuclease activity	5	46	60	10.86957	76.66666	3.739	0.007
17076	purine nucleotide binding	18	1369	1735	1.314828	78.9049	−2.845	0.007
5083	small GTPase regulator activity	11	182	173	6.043956	105.2023	3.211	0.009
8171	O-methyltransferase activity	2	8	9	25	88.88889	4.159	0.01
8108	UDP-glucose\:hexose-1-phosphate	1	1	1	100	100	6.352	0.012
	uridylyltransferase activity							
17124	SH3 domain binding	3	17	18	17.64706	94.44444	4.09	0.013
3712	transcription cofactor activity	13	274	314	4.744525	87.26115	2.538	0.014
8374	O-acyltransferase activity	3	23	33	13.04348	69.69697	3.32	0.017
8408	3′-5′ exonuclease activity	3	24	33	12.5	72.72727	3.218	0.017
48156	tau protein binding	1	1	2	100	50	6.352	0.018
50749	apolipoprotein E receptor binding	1	1	1	100	100	6.352	0.018
5355	glucose transporter activity	2	9	14	22.22222	64.28571	3.869	0.018
15085	calcium ion transporter activity	2	9	13	22.22222	69.23077	3.869	0.019
47066	phospholipid-hydroperoxide	1	1	1	100	100	6.352	0.021
	glutathione peroxidase activity							
3714	transcription corepressor activity	7	109	125	6.422019	87.2	2.734	0.021
50501	hyaluronan synthase activity	1	1	3	100	33.33333	6.352	0.022
16564	transcriptional repressor activity	10	194	236	5.154639	82.20339	2.503	0.022
4671	protein-S-isoprenylcysteine O-	1	1	1	100	100	6.352	0.023
	methyltransferase activity							
15149	hexose transporter activity	2	10	15	20	66.66666	3.62	0.023
15145	monosaccharide transporter	2	10	15	20	66.66666	3.62	0.023
	activity							
19968	interleukin-1\ II\, blocking binding	1	1	2	100	50	6.352	0.024
4910	interleukin-1\, II\, blockingactivity	1	1	2	100	50	6.352	0.024
5118	sevenless binding	1	1	2	100	50	6.352	0.025
26	alpha-1\,2-mannosyltransferase	1	1	1	100	100	6.352	0.025
4244	mitochondrial inner membrane	1	1	1	100	100	6.352	0.025
	peptidase activity							
45518	interleukin-22 receptor binding	1	1	1	100	100	6.352	0.026
15227	acyl carnitine transporter activity	1	1	1	100	100	6.352	0.026
5536	glucose binding	1	1	2	100	50	6.352	0.027
16209	antioxidant activity	3	27	46	11.11111	58.69565	2.944	0.027
15646	permease activity	1	1	1	100	100	6.352	0.028
15196	L-tryptophan transporter activity	1	1	1	100	100	6.352	0.028
15216	purine nucleotide transporter activity	1	1	1	100	100	6.352	0.028
51370	ZASP binding	1	1	1	100	100	6.352	0.029
51374	FATZ 1 binding	1	1	1	100	100	6.352	0.029
51373	FATZ binding	1	1	2	100	50	6.352	0.029
5088	Ras guanyl-nucleotide exchange	5	74	88	6.756757	84.09091	2.437	0.03
	factor activity							
30346	protein phosphatase 2B binding	1	1	2	100	50	6.352	0.034
42903	tubulin deacetylase activity	1	2	2	50	100	4.38	0.035
16409	palmitoyltransferase activity	2	12	14	16.66667	85.71429	3.214	0.036
46873	metal ion transporter activity	4	45	63	8.888889	71.42857	2.831	0.036
166	nucleotide binding	26	1577	1994	1.6487	79.08727	−2.154	0.036
30197	extracellular matrix constituent\,	1	2	3	50	66.66666	4.38	0.038
	lubricant activity							
900	translation repressor activity	1	2	2	50	100	4.38	0.039
42835	BRE binding	1	2	2	50	100	4.38	0.039
8451	X-Pro aminopeptidase activity	1	2	3	50	66.66666	4.38	0.04
30554	adenyl nucleotide binding	16	1109	1393	1.442741	79.61235	−2.233	0.04
4307	ethanolaminephospho-	1	2	2	50	100	4.38	0.042
	transferase activity							
15266	protein channel activity	1	2	2	50	100	4.38	0.045
8321	Ral guanyl-nucleotide exchange	1	2	2	50	100	4.38	0.046
	factor activity							
30275	LRR domain binding	1	2	2	50	100	4.38	0.047
15215	nucleotide transporter activity	1	2	2	50	100	4.38	0.048
15016	[heparan sulfate]-glucosamine N-	1	2	4	50	50	4.38	0.048
	sulfotransferase activity							
15082	di-\, tri-valent inorganic cation	3	30	43	10	69.76744	2.707	0.048
	transporter activity							
51393	alpha-actinin binding	1	2	3	50	66.66666	4.38	0.049
15173	aromatic amino acid transporter	1	2	2	50	100	4.38	0.049
3880	C-terminal protein carboxyl	1	2	2	50	100	4.38	0.049
	methyltransferase activity							
5128	erythropoietin receptor binding	1	2	3	50	66.66666	4.38	0.049
4908	interleukin-1 receptor activity	1	2	7	50	28.57143	4.38	0.049

3.2 Biological Process								
Go ID	GO Name	Genes	Genes	Genes	% Changed	% Present	Z Score	p Value
		Changed	Measured	In GO				
7369	gastrulation	5	42	51	11.90476	82.35294	4.009	0.004
50770	regulation of axonogenesis	4	27	30	14.81481	90	4.198	0.005
6349	imprinting	2	6	9	33.33333	66.66666	4.93	0.006
43547	positive regulation of GTPase	2	5	5	40	100	5.471	0.007
904	cellular morphogenesis during	8	117	172	6.837607	68.02325	3.128	0.008
	differentiation							
6635	fatty acid beta-oxidation	3	17	19	17.64706	89.47369	4.09	0.011
31175	neurite development	8	125	180	6.4	69.44444	2.914	0.011
40029	regulation of gene expression	4	41	47	9.756098	87.23404	3.064	0.012
16101	diterpenoid metabolic process	2	7	11	28.57143	63.63636	4.505	0.013
1523	retinoid metabolic process	2	7	11	28.57143	63.63636	4.505	0.013
7409	axonogenesis	7	102	150	6.862745	68	2.935	0.015
51281	positive regulation of release of	1	1	1	100	100	6.352	0.016
	sequestered calcium ion into cytosol							
51279	regulation of release of sequestered	1	1	1	100	100	6.352	0.016
	calcium ion into cytoplasm							
60042	morphogenesis in camera-type eye	1	1	1	100	100	6.352	0.016
42670	retinal cone cell differentiation	1	1	1	100	100	6.352	0.016
46549	retinal cone cell development	1	1	1	100	100	6.352	0.016
50767	regulation of neurogenesis	4	36	41	11.11111	87.80488	3.4	0.016
48667	neuron morphogenesis	7	107	158	6.542056	67.72152	2.79	0.016
48812	neurite morphogenesis	7	107	158	6.542056	67.72152	2.79	0.016
42461	photoreceptor cell development	2	8	12	25	66.66666	4.159	0.017
42462	eye photoreceptor cell development	2	8	11	25	72.72727	4.159	0.017
35023	regulation of Rho protein	5	65	76	7.692307	85.52631	2.776	0.017
	signal transduction							
50789	regulation of biological process	96	3196	4283	3.003755	74.62059	2.566	0.017
6807	nitrogen compound metabolic process	1	330	447	0.30303	73.8255	−2.541	0.017
9056	catabolic process	3	489	658	0.613497	74.31611	−2.659	0.018
7271	synaptic transmission\, cholinergic	2	8	11	25	72.72727	4.159	0.019
46530	photoreceptor cell differentiation	2	9	13	22.22222	69.23077	3.869	0.019
1754	eye photoreceptor cell differentiation	2	9	12	22.22222	75	3.869	0.019
18	regulation of DNA recombination	2	10	10	20	100	3.62	0.019
7506	gonadal mesoderm development	1	1	3	100	33.33333	6.352	0.02
122	negative regulation of transcription	7	121	153	5.785124	79.08497	2.424	0.02
	from RNA polymerase II promoter							
9308	amine metabolic process	1	306	416	0.326797	73.55769	−2.416	0.02
45911	positive regulation of DNA							
	recombination	1	1	1	100	100	6.352	0.021
19395	fatty acid oxidation	3	24	31	12.5	77.41936	3.218	0.021
	transforming growth factor beta							
7179	receptor signaling pathway	4	42	45	9.523809	93.33334	3.003	0.021
48856	anatomical structure development	47	1418	2087	3.314528	67.94442	2.356	0.021
31998	regulation of fatty acid beta-oxidation	1	1	1	100	100	6.352	0.022
	positive regulation of fatty							
32000	acid beta-oxidation	1	1	1	100	100	6.352	0.022
6312	mitotic recombination	2	7	7	28.57143	100	4.505	0.022
30092	regulation of flagellum biogenesis	1	1	1	100	100	6.352	0.024
45342	MHC class II biosynthetic process	1	1	5	100	20	6.352	0.024
	regulation of MHC class II							
45346	biosynthetic process	1	1	5	100	20	6.352	0.024
19372	lipoxygenase pathway	1	1	1	100	100	6.352	0.024
	positive regulation of							
45724	flagellum biogenesis	1	1	1	100	100	6.352	0.024
45347	negative regulation of MHC class II biosynthetic process	1	1	2	100	50	6.352	0.024
	hemopoietic progenitor							
2244	cell differentiation	1	1	1	100	100	6.352	0.025
2318	myeloid cell differentiation	1	1	1	100	100	6.352	0.025
48666	neuron development	8	144	204	5.555555	70.58823	2.467	0.025
32990	cell part morphogenesis	9	173	234	5.202312	73.93163	2.402	0.025
48858	cell projection morphogenesis	9	173	234	5.202312	73.93163	2.402	0.025
30030	cell projection and biogenesis	9	173	234	5.202312	73.93163	2.402	0.025
50794	regulation of cellular process	89	2977	3930	2.989587	75.75063	2.383	0.025
6844	acyl carnitine transport	1	1	1	100	100	6.352	0.026
6721	terpenoid metabolic process	2	10	14	20	71.42857	3.62	0.026
9720	detection of hormone stimulus	1	1	1	100	100	6.352	0.028
9726	detection of endogenous stimulus	1	1	2	100	50	6.352	0.028
48250	mitochondrial iron ion transport	1	1	1	100	100	6.352	0.028
6979	response to oxidative stress	4	54	70	7.407407	77.14286	2.392	0.028
44248	cellular catabolic process	3	415	521	0.722892	79.65451	−2.293	0.029
51056	regulation of small GTPase							
	mediated signal transduction	7	124	148	5.645161	83.78378	2.352	0.031
65007	biological regulation	100	3425	4645	2.919708	73.7352	2.31	0.031
45892	negative regulation of transcription	9	181	218	4.972376	83.02752	2.255	0.031
6519	amino acid and metabolic process	1	250	337	0.4	74.18398	−2.102	0.031
42632	cholesterol homeostasis	2	13	15	15.38461	86.66666	3.045	0.032
30218	erythrocyte differentiation	3	30	32	10	93.75	2.707	0.032
35295	tube development	6	96	112	6.25	85.71429	2.454	0.034
44255	cellular lipid metabolic process	17	418	567	4.066986	73.72134	2.237	0.034
51186	cofactor metabolic process	0	172	197	0	87.30965	−2.081	0.035
30324	lung development	3	32	43	9.375	74.4186	2.565	0.037
32502	developmental process	75	2485	3501	3.018109	70.97972	2.217	0.037
9790	embryonic development	12	262	330	4.580153	79.39394	2.305	0.038
32501	multicellular organismal process	63	2030	3635	3.103448	55.84594	2.229	0.039
302	response to reactive oxygen species	2	13	18	15.38461	72.22222	3.045	0.04
6644	phospholipid metabolic process	6	102	130	5.882353	78.46154	2.288	0.041
1880	Mullerian duct regression	1	2	3	50	66.66666	4.38	0.043
60033	anatomical structure regression	1	2	3	50	66.66666	4.38	0.043
48041	focal adhesion formation	2	13	12	15.38461	108.3333	3.045	0.043
7286	spermatid development	3	27	35	11.11111	77.14286	2.944	0.043
3007	heart morphogenesis	2	14	12	14.28571	116.6667	2.892	0.043
48515	spermatid differentiation	3	28	37	10.71429	75.67567	2.861	0.043
51262	protein tetramerization	2	15	18	13.33333	83.33334	2.753	0.043
43414	biopolymer methylation	3	33	39	9.090909	84.61539	2.499	0.043
19369	arachidonic acid metabolic process	1	2	5	50	40	4.38	0.044
30336	negative regulation of cell migration	2	13	15	15.38461	86.66666	3.045	0.044
6874	calcium ion homeostasis	4	53	111	7.54717	47.74775	2.436	0.044
6898	receptor-mediated endocytosis	3	32	44	9.375	72.72727	2.565	0.045
30323	respiratory tube development	3	34	45	8.823529	75.55556	2.435	0.045
9057	macromolecule catabolic process	2	318	406	0.628931	78.32513	−2.109	0.045
	establishment of polarity of							
42249	embryonic epithelium	1	2	1	50	200	4.38	0.047
31133	regulation of axon diameter	1	2	1	50	200	4.38	0.049
18410	protein carboxyl-terminal blocking	1	2	2	50	100	4.38	0.049
6481	C-terminal amino acid methylation	1	2	2	50	100	4.38	0.049
42157	lipoprotein metabolic process	4	56	66	7.142857	84.84849	2.307	0.049
43285	biopolymer catabolic process	1	253	313	0.395257	80.83067	−2.12	0.049

3.3 Cellular component								
Go ID	GO Name	Genes	Genes	Genes	% Changed	% Present	Z Score	p Value
		Changed	Measured	In GO				
5930	axoneme	3	11	19	27.27273	57.89474	5.368	0
16020	membrane	64	3636	5888	1.760176	61.75272	−3.174	0
44463	cell projection part	5	40	52	12.5	76.92308	4.158	0.001
178	exosome (RNase complex)	3	10	13	30	76.92308	5.68	0.002
43197	dendritic spine	2	7	8	28.57143	87.5	4.505	0.008
44425	membrane part	54	2931	4851	1.842375	60.42053	−2.38	0.016
791	euchromatin	1	1	1	100	100	6.352	0.017
42720	mitochondrial inner	1	1	1	100	100	6.352	0.025
	membrane peptidase complex							
783	nuclear telomere cap complex	1	1	1	100	100	6.352	0.026
782	telomere cap complex	1	1	1	100	100	6.352	0.026
31312	extrinsic organelle membrane	1	1	1	100	100	6.352	0.027
31314	extrinsic to mitochondrial	1	1	1	100	100	6.352	0.027
31314	inner membrane							
16021	integral to membrane	48	2605	4470	1.84261	58.2774	−2.197	0.027
31224	intrinsic to membrane	48	2610	4483	1.83908	58.21994	−2.214	0.027
30018	Z disc	2	12	19	16.66667	63.15789	3.214	0.032
5672	transcription factor TFIIA complex	1	2	2	50	100	4.38	0.04
9897	external side of plasma membrane	3	35	67	8.571428	52.23881	2.373	0.049

Taken together these results reveal that oxalate exposure differentially modulates genes required for molecular functions, biological pathways, and cellular components. Moreover, the number of genes up-regulated in each functional group is far less than the number of genes down regulated. Surprisingly, oxalate exposure significantly suppresses the expression of 552 genes, while only upregulating 105 genes associated with biological processes. These results show the molecular network of gene expression that is associated with cellular dysfunction modulated by oxalate.

### Oxalate exposure modulates gene expression by regulating transcription factors

To further gain insights into how oxalate controls differential gene expression, we analyzed the gene expression of transcription regulatory genes in response to oxalate exposure in HK2 cells. The results presented in Table 4 show the effects of oxalate exposure on the expression of genes involved in the regulation of transcription. These results show that oxalate exposure upregulates genes associated with the suppression of gene expression and at the same time down-regulates genes associated with transcription. Further assignment of these transcriptional regulatory genes by gene ontology groups (Table 4) reveals how oxalate influences gene expression of various transcription regulatory genes. These results reveal the selective nature of oxalate interactions with transcription regulatory machinery and provide a mechanistic link as to how oxalate treatment regulates changes in global gene expression in HK-2 cells.

**Table 4:** Genes involved in regulation of transcription in HK-2 cells after exposure to oxalate.

GO ID	GO Name	GO	Genes	Genes	Genes	% Changed	% Present	Z Score	p Value
		Type	Changed	Measured	in GO				
	**Up-Regulated**								
30528	transcription regulator activity	F	15	1012	1398	1.482213	72.38913	3.664	0
3700	transcription factor activity	F	10	630	936	1.587302	67.30769	3.182	0.006
3704	specific RNA polymerase II	F	2	31	41	6.451613	75.60976	4.139	0.016
	transcription factor activity								
16564	transcriptional repressor activity	F	4	194	236	2.061856	82.20339	2.578	0.032
6357	regulation of transcription from RNA	P	6	371	474	1.617251	78.27004	2.486	0.024
	polymerase II promoter								
45892	negative regulation of transcription\,	P	4	181	218	2.209945	83.02752	2.745	0.027
	DNA-dependent								
42994	cytoplasmic sequestering of	P	1	7	10	14.28571	70	4.604	0.041
	transcription factor								
42992	negative regulation of transcription	P	1	8	12	12.5	66.66666	4.279	0.048
	factor import into nucleus								
	**Down-Regulated**								
3712	transcription cofactor activity	F	13	274	314	4.744525	87.26115	2.538	0.014
3714	transcription co-repressor activity	F	7	109	125	6.422019	87.2	2.734	0.021
16564	transcriptional repressor activity	F	10	194	236	5.154639	82.20339	2.503	0.022
40029	regulation of gene expression	P	4	41	47	9.756098	87.23404	3.064	0.012
122	negative regulation of transcription	P	7	121	153	5.785124	79.08497	2.424	0.02
	from RNApolymerase II promoter								
45892	negative regulation of transcription\,	P	9	181	218	4.972376	83.02752	2.255	0.031
	DNA-dependent								
5672	transcription factor TFIIA complex	C	1	2	2	50	100	4.38	0.04

### Oxalate exposure modulates expression of gene associated with Signal transduction pathways

**Figure 3:**
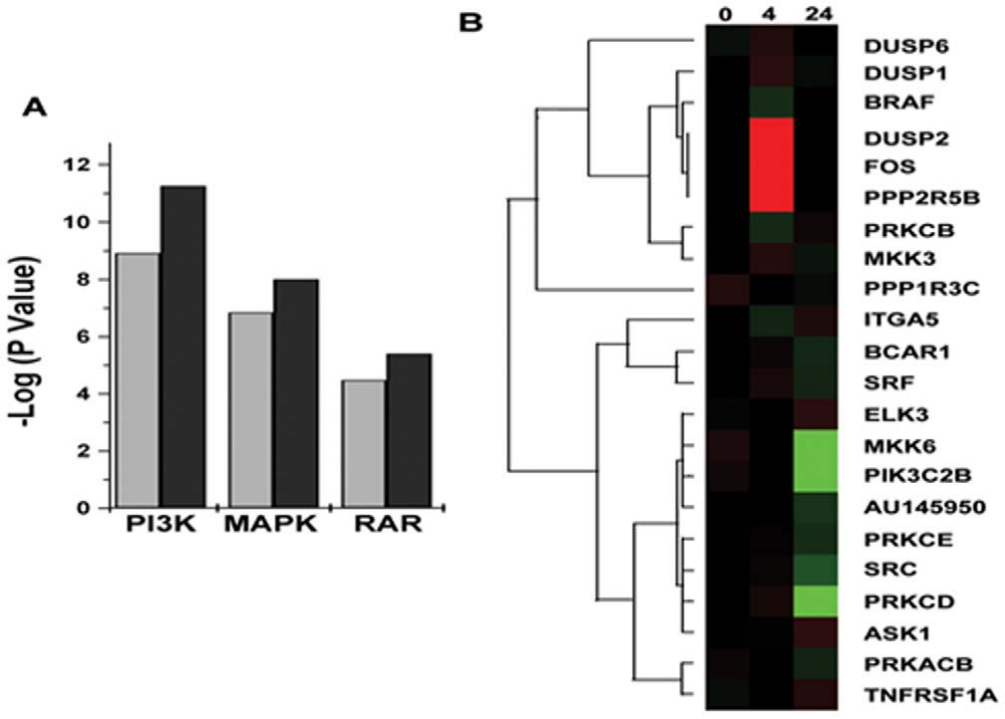
Regulation of Gene expression associated with Molecular functions, Biological Processes or Cellular component. Gene expression was analyzed with respect to gene ontology Groups and the number of genes that showed more than fivefold changes within each ontology group were identified. A)Genes that show an average change more than 5 fold up-regulation compared to control (p value<0.05) upon oxalate exposure grouped based on gene ontology using GenMAPP ; B) Genes that show an average change more than 5 fold down-regulation compared to control (p value<0.05) upon oxalate exposure grouped based on gene ontology using GenMAPP.

The results presented in Figure 3A show that several genes associated with signal transduction pathways, PI3 Kinase, MAP kinase and Retinoic acid Receptor pathways, show significantly altered expressions. In general, more genes associated with these three pathways are up-regulated in renal epithelial cells as early as 4 hours into oxalate exposure and some of these genes remain elevated for up to 24 hours. The results presented in Figure 3B show the heat MAP of this data specifically listing the genes associated with these pathways that are differentially regulated in HK2 cells upon exposure to oxalate.

### Genes that are always differentially expressed in renal cells after exposure to oxalate may serve as gene expression signature of oxalate nephrotoxicity

**Figure 4:**
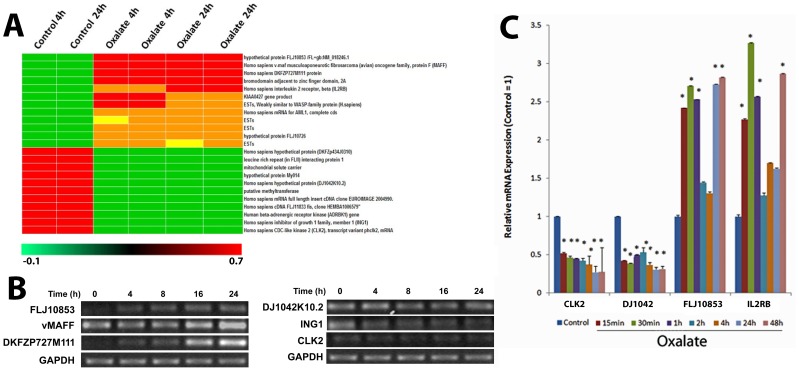
Gene Expression Signature of Oxalate Nephrotoxicity. (A) Gene expression changes highly significant in HK-2 cells exposed to oxalate compared to control cells and their fold changes. (B) HK-2 cells were treated with Oxalate for various time points and RT-PCR was used to identify differences in mRNA levels with GAPDH as a loading control. Left panel shows genes that are highly up-regulated and the right panel shows genes that are down-regulated upon oxalate exposure. C) Real Time Quantitative PCR analysis of time dependent changes in expression of two upregulated and two down regulated genes.

The results presented in figure 4 A shows a heat-map of set of twenty genes that are differentially modulated in HK-2 cells upon exposure to oxalate regardless of the duration of exposure. These include genes that are expressed either only in the oxalate treated HK-2 cells (absent in control) or only in control cells (absent in oxalate treated HK-2 cells). The expression of the differentially expressed genes was confirmed in parallel experiments using relative quantitative RT-PCR (Fig. 4B). We also evaluated the duration of expression of these genes in response to oxalate exposure. For these experiments we evaluated the quantitative changes by using real-time PCR. The results of these studies show a time dependent change in the mRNA levels of the genes tested [Fig. 4C]. These results also shows that these genes are turned on as early as 15 minutes into oxalate exposure and remain differentially expressed for a long period of time [over 48 hours]. Taken together these results suggest that transcriptional profiling using this set of genes may be useful to monitor oxalate nephrotoxicity.

## Discussion

Our studies describe genome wide changes in the transcript expression of renal epithelial cells in response to oxalate. Specifically, these studies show the upregulation of 750 genes and their transcripts and the down regulation of 2,276 genes and their transcripts that have not been reported before. More importantly, these studies identified the differential expression of genes that regulates transcription machinenary and thus may serve as drivers in oxalate induced transcriptional changes associated with oxalate nephropathy, including nephrolithiasis. Nephrolithiasis is a multi-factorial disorder which, in a majority of patients, results in renal deposition of calcium oxalate. While chronic hyperoxaluria is a common finding in patients with inherited metabolic conditions of primary-hyperoxaluria, a majority of patients generally show only a mild elevation in urinary oxalate. High urinary levels of oxalate, as seen in patients with hyperoxaluria, are generally associated with interstitial nephritis, extracellular fibrosis, and may eventually result in kidney failure if left uncorrected [28]. The development of a kidney stone depends on many extrinsic and intrinsic factors, which are not completely understood. The disease is multi-factorial and the nature of urine milieu is complex. As such, focusing on specific urinary components associated with urolithaisis is critical. Previous studies have shown that oxalate exposure to the renal epithelial cells promotes cellular dysfunction, which has been shown to promote renal crystal retention [5], [29]–[31]. Oxalate renal interactions are complex and poorly understood. While many previous studies have evaluated the over expression of a few genes in renal epithelial cells in response to oxalate, our results presented here profiled oxalate renal cell interactions in an unbiased fashion using microarray gene expression technology. Our studies revealed that in addition to promoting the expression of specific genes, oxalate exposure to renal epithelial cells is also associated with silencing the expression of many genes. We also show that oxalate differentially regulates the expression of genes in each ontology group: molecular functions, biological processes, and cellular components. Our studies further demonstrate that oxalate exposure differentially effects the expression of transcription regulatory genes, thus suggesting that the modulation of transcription regulatory genes might be a potential regulatory mechanism of modulating gene expression by oxalate. In addition, we identified a set of twenty genes differentially regulated by oxalate which might serve as a useful marker for monitoring oxalate nephrotoxicity.

Gene expression at any given time point represents a snapshot of a cells molecular machinery. Our results [Fig. 1A, B] show that oxalate exposure is associated with decrease in mRNA levels of several genes in all systems. These results show that oxalate exposure to the renal cells for 24 hours is associated with the net inhibition of the expression of 2,276 of genes. This unprecedented suppression of a large number of genes in renal epithelial cells suggests a program of the suppression of transcriptional activity. However, previous studies by have shown that the exposure of renal epithelial cells to oxalate results in the up-regulation of several genes [12], [15], [18], [20], [20], [22,23]. Thus, the notion of a general transcriptional repression is too simplistic of an explanation. Further analysis of our gene expression data (Fig. 1 C) reveals that although, over all, less genes are expressed in oxalate exposed cells for either 4 hour or 24 hour durations (26, 211 (control)vs. 25,107 (oxalate-4 h) and 23,935 (oxalate-24 hours), a number of genes (∼750 genes) are up-regulated. These findings demonstrate that oxalate exposure differentially and selectively regulates gene expression. Moreover, expression profiling at 4 hour and 24 hour intervals following oxalate exposure suggest that gene expression changes in response to oxalate are in part governed by the duration of exposure.

The studies presented here have identified changes in the expression of genes that are responsible for increased transcription, lending credibility to the earlier observations. HK-2 cells exposed to oxalate show extensive changes in gene expression over a wide spectrum of functions. In addition to genes that have been already implicated, many unique genes were found to be either expressed or inhibited upon oxalate exposure. We observed that some genes are differentially affected depending on the duration of oxalate exposure. HK-2 cells exposed to oxalate for 24 hours show differential gene expression changes compared to HK-2 cells exposed for only 4 hours. This suggests that in addition to the amount of oxalate present in the cell's vicinity, the duration of exposure to oxalate also plays an important role in modulating gene expression in renal cells following oxalate exposure.

Differential gene expression as a result of oxalate exposure can stimulate different responses in the cells directly exposed to oxalate that may ultimately lead to either survival or cell death depending on the concentration and the duration of exposure to oxalate. Results first reported by us [12], that have since been confirmed by several others, have shown that oxalate is toxic to renal epithelial cells. Analysis of the gene expression data using gene ontology software reveals that oxalate exposure inhibits the expression of over hundreds of genes that are required for cellular functions, which would suggest that oxalate might broadly impact cellular functions. Thus, renal cell dysfunction in response to oxalate may be in part driven by altered gene expression; however, the present study design does not permit the separation of causal and bystander genes. It is also possible that some of the changes in gene expression are causal, while other changes are bystander effects. In other words, whether the changes in gene expression play a causal role in oxalate nephrotoxicity or whether the gene expression changes are a result of dysfunction need to be addressed in additional studies.

Our analysis of gene expression data in renal epithelial ells in response to oxalate exposure revealed that 43 transcription regulatory genes are overexpressed while 51 transcription regulatory genes are down regulated by oxalate (Table 4). Given the enormous implications in regulation of cellular function by the modulation of gene expression, specific mechanisms are in place in eukaryotic cells that regulate gene expression. Transcription regulatory machinenary in eukaryotes involves specific transcription factors and transcription inhibitors; proteins that are required to turn on and turn off the expression of particular genes. These considerations point to a possible mechanism of how oxalate differentially regulates the gene expression of such a large number of genes.

Given that oxalate is a metabolic end product in humans that cannot be further metabolized, such large scale changes in gene expression in renal epithelial cells in response to high oxalate levels points to an indirect mechanism of action, which may involve the interaction of oxalate with the cell membrane or in intracellular components. The primary site of oxalate action in cell remains unknown. Irrespective of primary site of action, one of the most common means by which cells sense changes is by activating the signal transduction pathways, especially the stress signal pathways. The stress associated signals are transduced through a series of proteins that are activated by phosphorylation/dephosphorylation steps and are finally turned into transcription factors, causing changes in gene expression. Though the present study design does not allow for the identification of activity changes due to phosphorylation, we identified changes in the gene expressions of upstream activators of several signaling pathways. Proteins like Ras, Fas and MKK are highly up-regulated as a result of oxalate exposure. These proteins are known to play important roles in JNK/SAPK signaling and p38 MAPK signaling. These results are in agreement with previous studies, by us [14], [15], [ and 24] and others [32], that identified an active role for Stress Activated Protein Kinases in oxalate renal cell interactions. We also identified changes in the expression of genes associated with retinoic Acid Receptor Signaling Pathway. Clearly additional studies are required to evaluate the functional consequence of these gene expression changes.

In summary, our study is the first attempt at profiling the Genome-wide expression changes in human renal epithelial cells as a result of exposure to oxalate. Results from our study point to complex and intricate mechanisms, including differential gene expression, in renal epithelial cells in response to oxalate exposure. Clearly further studies are required to completely understand the implications of the plethora of changes in gene expression occurring as a result of oxalate exposure in renal epithelial cells. We must separate and characterize the genes that are derived from the by-stander effect and identify the genes whose altered expression is responsible for oxalate nephrotoxicity.

## Concise Methods

### Cell culture

Human Kidney Epithelial Cells, HK-2, were procured from ATCC and maintained in a DMEM medium supplemented with 10% Fetal Bovine Serum and antibiotics. Before Oxalate treatments, cells were serum starved for 16 to 20 hours. Media components were procured form Invitrogen Corporation and all other chemicals were procured from Sigma-Aldrich.

### Microarray analysis using Affymetrix Gene Chip

HK2 cells were incubated in the presence of 1 mM Sodium Oxalate for different amounts of time and all cellular RNA was isolated using a RNEasy Kit (Qiagen). RNA was quantified with a NanoDrop ND-1000 spectrophotometer and tested for quality using an Agilent Bioanalyzer 2100 (Agilent) before being used for microarray analysis. cRNA was prepared using 50 ng of RNA and then hybridized to an Affymetrix Human genome U133 Plus 2.0 gene array comprising of 11 independent replicate sets for each message Hybridization, staining, and post-hybridization washes were completed according to the manufacturer's recommendations (Affymetrix). Following hybridization, gene arrays were processed with a GeneChip fluidics station 450 and double staining was captured using a gene array scanner 3000. All experiments were designed to comply with the Minimum Information About a Microarray Experiment [MIAME; http://www.mged.org/index.html] guidelines and were interpreted by independent verification [33].

### Data analysis and bioinformatics

Hybridization intensities were quantified from the data image files using Gene Chip Operating Software algorithms (GCOS1.2, Affymetrix) with global scaling. Data analysis was performed using a Data Mining Tool [DMT 3.1, Affymetrix] and a GeneSpring 7.2 (Silicon Genetics). Cell Intensity files were processed into expression values for all the 55,000 probe sets (transcripts) on each array and following the respective normalization step. Differentially expressed genes were selected if they passed Welch's *t* test and parametric test (variance not assumed equal, *P*<0.05) and showed at least 2-fold changes between control and oxalate treated sets. Global gene expression was visualized by STAGE, a Smart Tool for Gene Expression analysis developed in house (Bhat S and Koul H). Differentially expressed genes were classified according to the Gene Ontology functional category (GenMAPP v2). Cluster and Heatmap images were generated using BRB-Array tools [34], freely available from http://linus.nci.nih.gov/BRB-ArrayTools.html.

### Reverse transcriptase PCR

1 µg RNA was used to synthesize cDNA by using iScript, a cDNA synthesis Kit [Bio-Rad Laboratories], and reverse transcriptase. PCR was performed with gene specific primers using Platinum Taq Polymerase (Invitrogen) and separating the products on a 1% agarose gel. Primers were procured from Integrated DNA Technologies and primer sequences used are described in Table 4.

### Real-time qPCR

Melt curve analysis was included to assure that only one PCR product was formed. Primers were designed to generate a PCR amplification product of 100–550 bp. Only primer pairs yielding unique amplification products without primer dimer formation were subsequently used for real-time PCR assays. Expression was related to the control gene [GAPDH], which did not change under any of the experimental conditions studied.

The real-time PCR reaction mixture was prepared in a Light Cycler 480 (Roche Diagnostics), a Multiwell 96-well plate containing 10 µM of each primer, 10 µl of 2× master mix, and 1 µl of cDNA template in a final reaction volume of 20 µl. The real-time PCR amplification was performed using the specific primers as shown in Table 1, using the following cycle parameters: enzyme activation at 95°C for 10 min; 45 cycles of 95°C for 10 s, 63°C for 10 s and 72°C for 10 s. Following the amplification phase, a cooling step was performed at 4°C for 10 s (ramp rate of 1.5°C/s). Acquisition of the fluorescence signal was performed using the Mono Hydrolysis Probe setting [483–523 nm] following the 72°C extension phase of each cycle. GAPDH primers were included to normalize variation from sample to sample. All experiments were repeated three times using three independent preparations of cDNA.
